# Green Fluorescent Protein Labeling of *Listeria*, *Salmonella*, and *Escherichia coli* O157:H7 for Safety-Related Studies

**DOI:** 10.1371/journal.pone.0018083

**Published:** 2011-04-04

**Authors:** Li Ma, Guodong Zhang, Michael P. Doyle

**Affiliations:** 1 Department of Entomology and Plant Pathology, National Institute for Microbial Forensics and Food and Agricultural Biosecurity, Oklahoma State University, Stillwater, Oklahoma, United States of America; 2 Center for Food Safety and Applied Nutrition, Food and Drug Administration, College Park, Maryland, United States of America; 3 Center for Food Safety, University of Georgia, Griffin, Georgia, United States of America; Tulane University, United States of America

## Abstract

Many food safety-related studies require tracking of introduced foodborne pathogens to monitor their fate in complex environments. The green fluorescent protein (GFP) gene (*gfp)* provides an easily detectable phenotype so has been used to label many microorganisms for ecological studies. The objectives of this study were to label major foodborne pathogens and related bacteria, including *Listeria monocytogenes, Listeria innocua*, *Salmonella,* and *Escherichia coli* O157:H7 strains, with GFP and characterize the labeled strains for stability of the GFP plasmid and the plasmid's effect on bacterial growth. GFP plasmids were introduced into these strains by a CaCl_2_ procedure, conjugation or electroporation. Stability of the label was determined through sequential propagation of labeled strains in the absence of selective pressure, and rates of plasmid-loss were calculated. Stability of the GFP plasmid varied among the labeled species and strains, with the most stable GFP label observed in *E. coli* O157:H7. When grown in nonselective media for two consecutive subcultures (ca. 20 generations), the rates of plasmid loss among labeled *E. coli* O157:H7, *Salmonella* and *Listeria* strains ranged from 0%–30%, 15.8%–99.9% and 8.1%–93.4%, respectively. Complete loss (>99.99%) of the plasmid occurred in some labeled strains after five consecutive subcultures in the absence of selective pressure, whereas it remained stable in others. The GFP plasmid had an insignificant effect on growth of most labeled strains. *E. coli* O157:H7, *Salmonella* and *Listeria* strains can be effectively labeled with the GFP plasmid which can be stable in some isolates for many generations without adversely affecting growth rates.

## Introduction

Studies of the behavior (persistence, survival, and colonization) of bacteria in their natural habitants have become very important because many traits are exhibited only in these natural settings so must be assessed in such environments to gain insight that have practical or scientific relevance. These natural habitants, can be a fermented food system, an animal's digestive tract, a plant's surface, soil, or a biofilm, are also microbiologically complex ecosystems with a large number of indigenous microbes. In these ecosystems, simplistic, rapid, precise, and sensitive detection methods are needed to track and distinguish inoculated bacteria from the indigenous microbial population. A popular method for labeling microbes is using green fluorescent protein (GFP). GFP, produced by the jellyfish *Aequorea victoria*, was originally partially characterized by Morise et al. in 1974 [1] and its gene (*gfp*) was isolated, cloned, and subsequently expressed in both eukaryotic and prokaryotic hosts [2], [3]. GFP emit in the jellyfish green light (excitation wavelength, 396 nm; emission wavelength, 508 nm) on energy transfer from aequorin, a Ca^2+^-activated photoprotein. The cloned GFP can be excited using light of a specific wavelength (396 nm) and emit green fluorescence light. Subsequently, the wild-type *gfp* gene has been mutated to many forms to improve detection and expression of the fluorescent protein in eukaryotic and prokaryotic hosts [4].

A major application of GFP is as a fusion reporter gene for use as a cytological marker to study gene expression or to monitor protein localization in live cells [2]. Another application is as a marker for tracking bacterial cells in ecological studies [5]–[8]. GFP-labeled bacteria have been used in monitoring either single cells or a cell population in survival studies of *E. coli* in aquatic environments [8], in elucidating the spatial arrangement of *Streptococcus* species in biofilm structure [5], in tracing *Vibrio parahaemolyticus* in oysters [6], and in investigating survival and spatial location of *Salmonella* in alfalfa sprouts [7]. GFP can be detected easily by fluorescence colony counting under long wavelength UV light, epifluorescence microscopy, laser confocal microscopy, and flow cytometry for individual cells or direct fluorescence measurement. Furthermore, the GFP phenotypes are detectable in all growth phases of bacteria, even under starvation conditions and in the viable but nonculturable state [9], [10].

Several potential problems can be associated with using labeled bacteria, including GFP, in ecological studies. First is the stability of the label. Investigators should be able to distinguish between loss of the label and loss of viability of the introduced strain from the test system. Secondly, it should be determined if expression of the label poses an additional metabolic burden that may affect the survival/metabolism of the labeled strain and may not provide an accurate representation of the behavior of the wild-type strain. A number of studies involving GFP-labeled strains of bacteria have revealed that GFP expression does not alter the biochemical, morphological, or survival characteristics of the labeled bacteria; however, only limited data regarding the effects of GFP expression on microbial growth are provided in these studies [11], [12]. The purposes of this study were to label strains of *E. coli* O157:H7, *Salmonella* Enteritidis, *Salmonella* Newport, *Listeria monocytogenes*, and *Listeria innocua* with *gfp*-containing plasmids for our food safety research projects and assess the stability of the GFP plasmid in the labeled bacteria and its effect on bacterial growth.

## Materials and Methods

### Bacterial strains, plasmids, and growth conditions

The bacterial strains and plasmids used in this study are listed in Table 1. *E. coli* JM 109p and *E. coli* HB101p were used as hosts to maintain *gfp*-containing plasmids pGFPuv and pNF8, respectively, whereas all the other strains were the recipients of the GFP plasmids. *E. coli* and *Salmonella* strains were grown at 37°C with agitation (100 rpm) in Luria-Bertani (LB) and Tryptic soy (Difco, Becton Dickinson, Sparks, MD), either broth or agar, respectively. *Listeria* strains were cultured at 37°C in brain heart infusion (BHI), either broth or agar (Difco Laboratories, Detroit, MI). When needed, the culture medium was supplemented with antibiotics at the following concentrations: ampicillin, 100 µg/ml (for *E .coli* O157:H7 strains) or 150 µg/ml (for *Salmonella* strains) or erythromycin, 8 µg/ml (for *Listeria* strains) or 100 µg/ml (for *E. coli* HB 101p). Modified Oxford agar (MOX) supplemented with erythromycin was used as the selective medium for *gfp*-labeled *Listeria* strains after conjugation (see below). All the antibiotics were purchased from Sigma (St. Louis, MO). All strains were stored in Microbank cryogenic vials (Prolab Diagnostics, Austin, TX) at −80°C for long-term preservation and maintained at 4°C on TSA slants with (*gfp*-containing strains) or without (strains without *gfp)* antibiotic supplement for short-term storage. Plasmids (pGFPuv and pNF8) were extracted from *E. coli* using the alkaline lysis method described by Sambrook et al. [13].

**Table 1:** Bacterial strains and plasmids used in this study.

Strain or plasmid	Relevant characteristic(s)	Reference or sources^a^
Bacterial strains		
* E. coli* HB101p	F^-^, *rec*A 13, pNF8 (Em^r^), pRK24 (Ap, Tc, Mob+)	Fortineau et al. [17]
* Salmonella* Enteritidis		
Benson 1		CFS
H3353		CFS
H4639		CFS
ME-18		CFS
* Salmonella* Newport		
11590-K	Beef isolate	CDC
* E.coli* O157:H7		
ATCC43888	Human feces, no stx1&2	FDA
C0283	Cattle feces	CFS
C7927	Human isolate (cider outbreak)	CFS
CV261	Cattle isolate, France, no stx1&2	FDA
CV267	Cattle isolate, France, no stx1&2	FDA
E0018	Calf feces	CFS
E0122	Cattle	CFS
E0144	Meat	CFS
F4546	Alfalfa sprout outbreak	CFS
H1730	Lettuce	CFS
K3995	Spinach outbreak, 2006	CDC
K4492	Taco bell outbreak, 2006	CDC
K4830	Taco John outbreak, 2006	CDC
Sea-13B88	Orange juice	CDC
* Listeria monocytogenes*		
101M	Beef and pork sausage isolate	USDA, ARS
12443	Monkey clinical isolate	CDC
51779	Belgium cheese	ATCC
F6854	Frankfurter isolate	USDA, ARS
F6900	Human clinical isolate, sporadic	CDC
G3982	Clinical isolate	CDC
H7550	Human clinical isolate, epidemic	CDC
J0161	Human clinical isolate, epidemic	CDC
Jalisco	Jalisco cheese	Kraft Inc.
ScottA	Human clinical isolate, epidemic	CDC
* Listeria innocua*		
18	Food processing environment	Silliker
24	Food processing environment	Silliker
33090	Cow brain	ATCC
E	Food processing environment	Silliker
Strep	Food processing environment	Silliker
Plasmids		
pGFP_uv_	Ap^r^, P*lac*Ω *gfp*-uv	Clontech
pNF8	Em^r^, Mob+ (Inc P), *Pdlt*Ω*gfp*-*mut*1	Fortineau et al. [17]

a CDC, Centers for Disease Control and Prevention; FDA, Food and Drug Administration: CFS, Center for Food Safety, The University of Georgia; USDA, The United States Department of Agriculture.

### Construction of GFP-expressing *Salmonella* and *E. coli* O157:H7

The plasmid pGFPuv, purchased from Clontech Laboratories Inc. (Mountain View, CA), encoding a variant of GFP that emits brighter fluorescence when excited by standard UV light than GFP [14], was used to label *Salmonella* and *E. coli* O157:H7 strains. Since high ampicillin resistance (>100 µg/ml) of the strain to be GFP-labeled would interfere with selection of labeled transformants and possibly affect maintenance of the label in the presence of selection pressure, the ampicillin resistance profile for each strain to be labeled was determined by streaking fresh overnight culture onto a TSA plate supplemented with ampicillin (100 µg/ml or 150 µg/ml) using a semiquantitative scoring technique called ecometric evaluation [15], [16]. Only strains having absolute growth indices (AGIs) equal or less than 2 were selected for transformation. Competent *Salmonella* or *E. coli* O157:H7 cells were prepared and transformed with pGFPuv by the CaCl_2_ method described by Sambrook et al. [13]. Briefly, the competent cells were prepared by collecting a fresh culture of bacteria by centrifugation (3000×g, 10 min, 4°C), washing the cell pellet once with ice-cold 100 mM CaCl_2_ , and resuspending in the same solution at 1/25 original volume, while the transformation was carried out by mixing the competent cells with plasmid DNA (on ice for 10 min) before exposing the mixture to heat shock at 42°C for 90 sec in a water bath. Transformants were selected by plating on TSA supplemented with Amp (TSA+Amp, 100 µg/ml or 150 µg/ml) and colonies (3–5) that exhibited bright green fluorescence under UV light (396 nm) were restreaked for purification. The expression and uniformity of the GFP label among the cells was determined with an epifluorescent microscope (Olympus BH2-RFCA, Olympus America Inc., Lake Success, NY). GFP-labeled isolates (2–3) of each parental strain that exhibited bright and uniform green fluorescence both as colonies and individual cells were preserved at −80°C for further characterization later.

### Construction of GFP-expressing *Listeria* by conjugation

The plasmid pNF8, obtained from Fortineau et al. [17], a shuttle vector carrying the *gfp-mut*1 gene [4] that is transcribed from a strong and constitutive *L. monocytogenes* promoter *Pdlt*, was used to label the *Listeria* strains. *E. coli* strain HB101p carrying the conjugative helper plasmid pRK24 and pNF8 was used as the donor to transfer *gfp-mut*1 to *Listeria* recipients. Conjugation was carried out using a filter-mating method [18]. *E. coli* HB101p was grown in LB broth supplemented with 150 µg/ml erythromycin, whereas *Listeria* strains were grown in BHI broth. Both cultures were harvested at mid-log phase, which was determined spectrophometrically to be an optical density of ca. 0.5 at a wavelength of 600 nm (OD_600_), and washed twice with fresh BHI broth before combining at a ratio of 1∶2 (Donor *E. coli* HB101p: Recipient *Listeria*). The number of donors and recipients was determined by serial dilution and plating of the mixture on LB agar supplemented with erythromycin (donors) and MOX (for recipients). The mixture was filtered onto a washed HA-type filter (0.45 µm, Millipore, Bedford, MA) and the filter was washed once with BHI broth, then transferred onto a BHI agar plate and incubated overnight at 37°C. Cells on the filter were gently washed off the filter with 2.0 ml of BHI broth by pipeting. Portions (100 µl) of the suspension were spread plated onto MOX plates supplemented with 8 µg erythromycin/ml. GFP-labeled *Listeria* colonies (transconjugants containing pNF8), which were black colonies exhibiting green fluorescence under UV light, were enumerated. One to three green fluorescent colonies for each parental strain were restreaked onto MOX plates supplemented with 8 µg erythromycin/ml for purification. Transformation frequency of the conjugation is expressed as the number of transconjugants per output donor. Conjugation was performed on all *Listeria* strains and those that were not labeled with GFP after exposure to a variety of mating parameters (both donor and recipient cells' age, washing times before mating, mating ratio, and incubation time on filter) were subjected to electroporation (described below).

### Construction of GFP-expressing *Listeria* by electrotransformation

Electrotransformation of *Listeria* with pNF8 was initially performed on *L. monocytogenes* 12443 to optimize the electrotranformation procedures. Parameters tested included growth phase (mid-log phase, OD_600_ of 0.5 and late log phase, OD_600_ of 1.0), penicillin treatment (10 µg/ml) during cell growth, and different wash/electroporation buffers, either 10% glycerol or a sucrose magnesium phosphate buffer (SMP) consisting of 272 mM sucrose, 1 mM MgCl_2_, and 7 mM sodium phosphate pH 7.4. In general, penicillin (10 µg/ml) was added into a freshly growing culture of *L. monocytogenes* 12443 either at mid- or late log phase. Cells were grown for one additional hour after the addition of penicillin. The bacterial cells were then harvested, washed three times in an equal volume of cold wash/electroporation buffer, and suspended in the same buffer at a volume of 100-fold concentrated. For electroporation, 100 ul of the cell suspension was mixed with 0.2 µg of plasmid DNA. The sample in a 0.2-cm cuvette was subjected to a 2.25-kV, 200 Ω, 25-uF electric pulse by using a Gene Pulser and a Pulse Controller apparatus (Bio Rad Laboratories, Hercules, CA). BHI broth (1 ml, ice-cold) was added immediately to the curvette after the pulse, and the cells were incubated for 2 h at 37°C with agitation (100 rpm) before being plated on BHI agar supplemented with erythromycin (8 µg/ml). For each parameter tested, at least two independent transformation assays were performed. Transformation efficiencies were expressed as the number of transformants per micrograms of plasmid DNA. The optimal electrotransformation procedure, which was growing cells to late log phase with the addition of penicillin and using SMP as the wash/electroporation buffer, was determined and was used subsequently for electrotransformation of the *Listeria* strains.

### Plasmid stability testing

The stability of *gfp*-containing plasmids in GFP-labeled strains was determined under nonselective conditions. One to three GFP-labeled isolates derived from each parental strain were tested to assess stability variation among several sets of isolates. Overnight cultures of GFP-labeled strains grown in the presence of antibiotics were used to inoculate (1∶1000 dilution) broth (TSB for *E. coli* O157:H7 and *Salmonella*, and BHI for *Listeria*) without antibiotics. Bacteria were grown in broth without the presence of antibiotics and were transferred daily (24-h intervals) at a 1∶1000 dilution into fresh medium for 5 consecutive days. At 2 and 5 days, culture samples were diluted and spread onto agar plates without antibiotics (TSA for *E. coli* O157:H7 and *Salmonella*, and BHIA for *Listeria*). The plates were incubated until colonies appeared, and the numbers of total and green fluorescent colonies were determined. Under these growth conditions, cultures reached approximately 10^8^–10^9^ CFU/ml, which corresponded to approximately 10 generations per transfer using the formula: the number of generations  =  [(log cells at the end of incubation)-(log cells at the beginning of inoculation)]/0.301, the cell number at the beginning of inoculation was 10^5^–10^6^ CFU/ml (ave. 5.5 log), whereas at the end of incubation was 10^8^–10^9^ CFU/ml (ave. 8.5 log). Plasmid loss was calculated as the ratio between the plate counts of green fluorescent colonies and total counts on nonselective plates.

### Plasmid burden testing

Growth comparison between GFP-labeled strains and their corresponding parental strains was used to determine the metabolic burden of GFP-expressing plasmids on the GFP-labeled strains. The test strains from fresh overnight cultures were inoculated at a 1∶1000 dilution in their corresponding broth media without antibiotics. The inoculated cultures were incubated at 37°C with agitation (100 rpm). Bacterial growth was monitored spectrophometrically at OD_600_, which was determined at regular time intervals until 24 h of growth. Viable counts were also determined at regular time intervals by preparing serially diluted (1∶10) sample in phosphate-buffered saline (PBS, pH 7.2) and plating the appropriate dilutions on their corresponding nonselective media. Plates were incubated at 37°C for 24 h and the numbers of total and green fluorescent colonies were counted. Maximum growth rates were calculated from linear regression analysis of the linear portions of growth curves.

### Statistical analysis

Each experiment (burden tests) was conducted at least twice, and the Student *t* test was used to determine significant differences (*P*<0.05) between the maximum growth rates of GFP-labeled and parental strains.

## Results and Discussion

### Construction of GFP-expressing *Salmonella* and *E. coli* O157:H7 strains

Ampicillin resistance profiling of bacterial strains to be labeled by pGFPuv revealed that two *S*. Newport strains and one *E. coli* O157:H7 strain (H1730) were highly resistant to ampicillin (AGI = 5). Therefore, there was no attempt to label these three strains as the background colonies would be too high to allow efficient selection of GFP-labeled isolates on selective plates. Most of the *E. coli* O157:H7 and *Salmonella* strains were easily labeled with pGFPuv by the CaCl_2_ method. In general, higher transformation frequencies were observed in *E. coli* O157:H7 (9×10^2^ to 1.25×10^4^ transformants/µg) than in *Salmonella* (1.2×10^2^ to 1.4×10^3^ transformants/µg). However, GFP labeling of *E. coli* O157:H7 CV261 was not achieved by this method despite several attempts. The GFP-labeled strains were ampicillin resistant and appeared bright green when viewed under a UV light. Colonies of GFP-labeled and parental strains had identical morphological characteristics on TSA plates, except that some GFP-labeled colonies appeared light green or yellow when observed visually under normal room light. The CaCl_2_ method was desirable for labeling Gram-negative bacteria because it is easy and rapid to perform without the use of special equipment and produces sufficient transformants for selection. However, the antibiotic resistance profile of the strain to be labeled should be determined before transformation because an inherent resistance of the microbe may reduce the selective effect of the antibiotic and thereby not enable recovery of labeled strains, especially when there are low transformation frequencies.

### Construction of GFP-expressing *Listeria* strains

GFP labeling of *Listeria* by conjugation occurred readily for several strains; however, only five of fifteen *Listeria* strains were labeled (Table 2), hence, conjugation was a strain-specific event. It is noteworthy that in labeling of *Listeria* strains by conjugation, instead of using recipient *Listeria* strains with spontaneous antibiotic resistance (other than erythromycin) for counter selection against the donor *E. coli* after conjugation, we used the existing *Listeria* selective medium MOX supplemented with erythromycin as the recovery medium on which only listerial transconjugates containing pNF8 could grow. The same strategy can be applied to any bacteria with an existing selective medium in their conjugation experiments instead of selecting spontaneous antibiotic-resistant recipient cells for counterselection purposes. One advantage of our practice is avoiding the use of spontaneous antibiotic-resistant recipients because they are not the true original wild-type strains and may exhibit unexpected pleiotropic phenotypes [19]. Electrotransformation was chosen subsequently to label the remaining *Listeria* strains. A survey of relevant literature revealed that several important parameters should be evaluated initially to optimize electroporation efficiency for *Listeria* [20]. These factors include cell growth phase (middle vs. late log phase), cell wall- weakening agent treatment (penicillin, 10 µg/ml) during cell growth, and cell wash/electroporation buffers (either 10% glycerol or SMP). *L. monocytogenes* 12443 was used to evaluate these parameters and the results are summarized in Table 3. Without the addition of the cell wall-weakening agent, penicillin, mid-log phase cells (OD_600_ of 0.5) were most susceptible to electroporation, resulting in higher transformation frequencies than late-log phase cells (OD_600_ of 1.0). However, treatment of cells with penicillin (10 µg/ml) increased transformation frequencies in both phases, with major improvement (100-fold) for late-log phase cells. Therefore, late-log phase cells treated with penicillin was used to prepare all *Listeria* cells for electroporation. The most commonly used wash and electroporation buffers are 10% glycerol and SMP, and our results revealed that SMP outperformed 10% glycerol, likely because SMP is a better osmotic stabilizer and bridge between the cells and plasmid DNA. With the use of both conjugation and electroporation, all listerial strains were labeled with pNF8. Solely based on this study, electrotransformation is the preferred universal labeling procedure for labeling of *Listeria* strains with *gfp* when compared to conjugation. However, in situations other than this study, conjugation and electrotransformation should be complementary to each other, as direct imported plasmid DNA, such as those generated in electroporation, is often subjected to restriction/modification by the host systems which in turn limits the success of transformation, whereas the single-stranded DNA intermediates, such as those generated during conjugation, are not substrates for most restriction enzymes in the host systems. The GFP-labeled *Listeria* strains were resistant to erythromycin and appeared bright green when viewed under UV light; however, some colonies (usually larger) that did not fluorescence green also were present on selective medium containing erythromycin.

**Table 2:** Transformation frequencies of pNF8 from *E. coli* HB101p to *Listeria* strains.

Recipient strain	Transfer Frequency^a^
*L. monocytogenes* 101M	5.6×10^−6^
*L. monocytogenes* F6854	7.1×10^−7^
*L. innocua* 18	4.3×10^−6^
*L. innocua* 24	3.4×10^−6^
*L. innocua* E	2.5×10^−6^
Other *Listeria* (10 strains)	<1.0×10^−8^

a Transfer frequencies were means of two independent experiments and were expressed as the number of transconjugants per output donor colony-forming units.

**Table 3:** Factors influencing transformation frequency of *L. monocytogenes* 12443 electroporated with pNF8.

Growth phase	Penicillin treatment W/E buffe^a^	Transformation frequency^b^
Mid-log	SMP	1.0×10 ^3^
	Penicillin/SMP	3.0×10 ^3^
Late-log	SMP	1.5×10 ^2^
	Penicillin/SMP	1.1×10 ^4^
	Glycerol	2.0×10 ^1^
	Penicillin/Glycerol	3.2×10 ^1^

a Details provided in Materials and Methods section, W/E buffer is washing/electroporation buffer.

b Transformation frequency was calculated as numbers of transformants per microgram of plasmid DNA and the values are means of two independent experiments.

### Plasmid stability

One of the principal concerns raised regarding the use of a marker gene is the stability of the marker (plasmid). Plasmid stability tests, i.e., determining the proportion of plasmid-bearing cells remaining after a specified period of time, were conducted by culturing the GFP-labeled strains in the absence of antibiotic selection for ca. 50 generations. The results are summarized in Table 4. In general, the most stable GFP-labeled strains were *E. coli* O157:H7, followed by *Salmonella*, and then *Listeria*. Although some strains (*S*. Enteritidis Benson 1 and *L. innocua* 24) lost the *gfp*-containing plasmid quickly, most labeled strains were stable for ca. 20-generation passages without selective pressure, but many lost their *gfp*-containing plasmids (more than 1 log reduction) after ca. 50 generations. Plasmid stability or maintenance is primarily dependent on its segregational stability in which the plasmid must propagate in pace with the doubling rate of their host cells to be stably maintained in a cell population. The stability test was conducted under optimal growth conditions for the labeled strains which generated a much higher cell doubling rate than in most environmental situations in which the labeled strain is intended to be used. Hence, the GFP label is likely to be more stable in host cells under conditions that are less favorable for growth. This has been observed by Qazi et al. [21] with GFP-labeled (plasmid-borne) *L. monocytogenes*, who determined plasmid loss in the labeled strain was 50% after 8–10 generations of growth in broth but the ratio of fluorescent to non-fluorescent cells in a co-infection study in cell culture remained the same during the infection period, indicating the *gfp*-bearing plasmid was stable during intracellular growth. In our laboratory, GFP-labeled *L. innocua* and *L. monocytogenes* were used in aerosol studies [22] in which the green fluorescent phenotype was consistently retained during aerosolization either in a laboratory bioaerosol chamber or a pilot food-processing facility and through the duration of up to 3 h of the studies. When used in a study of the fate of foodborne pathogens in manure composts by our research group, both GFP-labeled *E. coli* O157:H7 and *L. innocua* were detected on the surface of the compost 56 days after inoculation [23]. Also, GFP-labeled *E. coli* O157:H7 strains were detected on the surface of lettuce leaves at least 26 days after leaf surface inoculation in an environtron [24].

**Table 4:** Plasmid stability in GFP-labeled bacterial strains.

GFP-labeled strain	Plasmid loss^a^ (%)	
	20 generations	50 generations
*S.* Enteritidis		
Benson 1	99.9	100^b^
H3353	64.5	91.9
H4639	77.0	99.0
ME-18	14.8	61.8
*S.* Newport		
11590-K	44.3	99.0
*E. coli* O157:H7		
ATCC43888	0.0	0.0
C0283	0.0	36.4
C7927	0.0	0.0
CV267	0.0	0.0
E0018	7.6	14.0
E0122	0.0	0.0
E0144	0.0	0.0
F4546	0.0	0.0
K3995	22.0	53.0
K4492	30.0	96.5
K4830	0.0	7.0
Sea-13B88	0.0	0.0
*L. monocytogenes*		
101M	31.7	95.3
12443	42.5	99.9
51779	38.7	99.4
F6854	52.0	99.9
F6900	25.0	99.9
G3982	8.2	77.1
H7550	45.3	96.8
J0161	24.1	96.4
Jalisco	25.9	99.8
Scott A	59.5	91.6
*L. innocua*		
18	47.6	89.6
24	93.4	99.9
33090	38.6	98.0
E	32.6	99.8
Strep	43.2	98.6

a Plasmid loss was calculated as the ratio between the counts of green fluorescent colonies and total colony counts on nonselective plates.

b Plasmid loss was ≥4 logs.

### Plasmid burden

**Figure 1:**
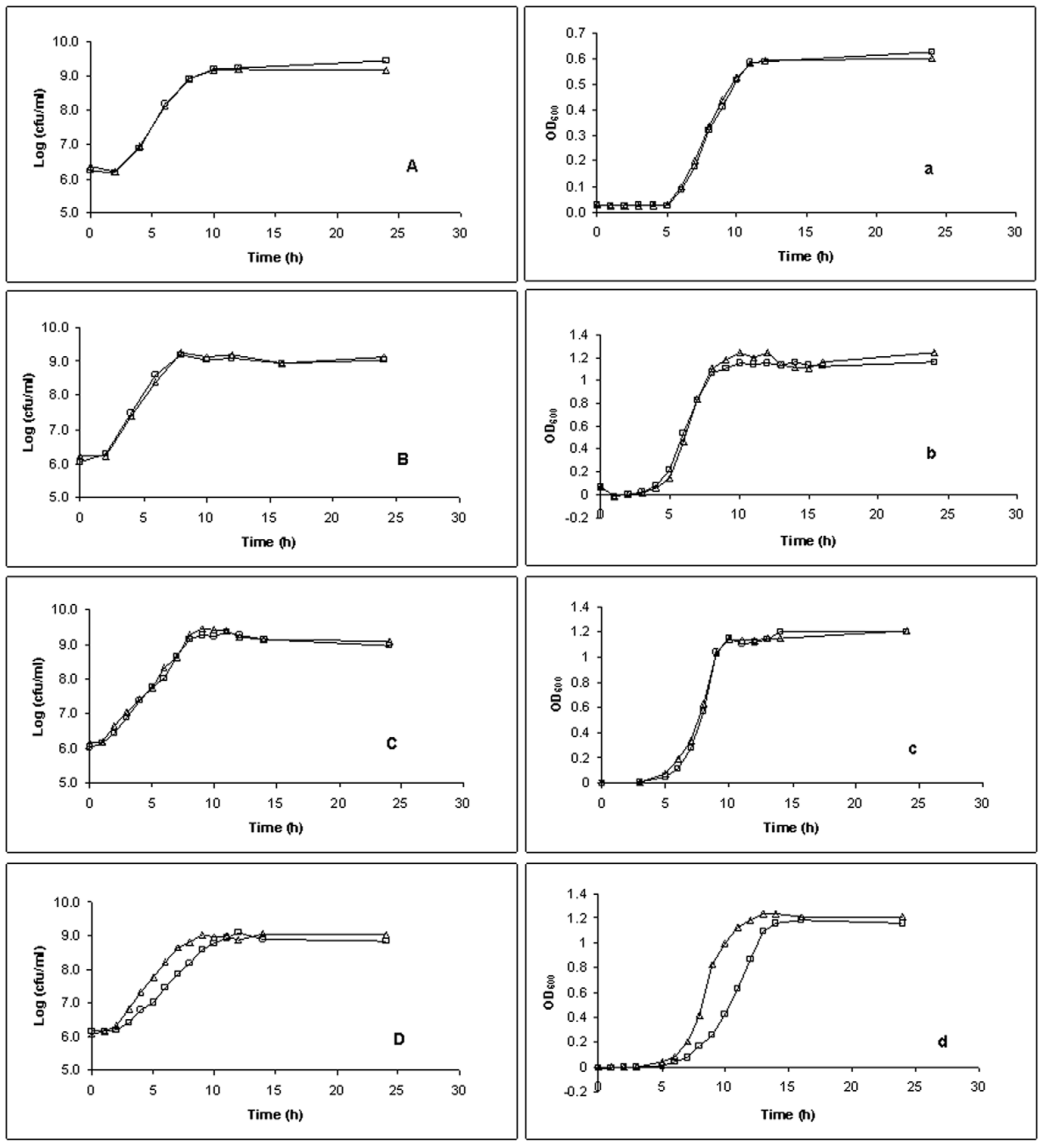
Comparison of growth rates of GFP-labeled and parental bacterial strains. Growth rates of GFP-labeled (Δ) and parental (□) bacterial strains were determined by cell counts (A, B, C, D) and spectrophotometrically at OD_600_ (a, b, c, d) for *E. coli* O157:H7 F4546 (A, a); *Salmonella* Enteritidis ME-18 (B, b); *Listeria innocua* E (C, c); and *Listeria monocytogenes* G3982 (D, d). Point values are the means of at least two trials.

Another concern regarding the use of a marker gene in bacterial behavior and physiology studies is that the addition of the marker gene and the high-level expression of the marker genes may result in substantial metabolic burden on the cells. Growth curves of both GFP-labeled and their parental strains were constructed from both plate counts and spectrophometric determinations and compared. The bacterial exponential growth phase was identified visually as the linear portion of the growth curve and the maximum growth rate of each was calculated by linear regression analysis of this linear portion by using at least four data points. For all 10 isogenic pairs of *E. coli* O157:H7, 4 pairs of *Salmonella*, 5 pairs of *L. innocua*, and 7 of 10 isogenic pairs of *L. monocytogenes* tested, nearly identical growth curves, either by OD_600_ readings or plate counts, were observed between GFP-labeled and their parental strains (Fig. 1). Lag periods, time of entry into the stationary phase, and final cell numbers and densities for each pairing were identical, with no significant differences in the maximum growth rates (µ_max_) of each pair. These results are in agreement with previous studies [25], [26] and indicate that the GFP label did not interfere with the growth characteristics of these host cells under optimal growth conditions, even though there is high-level constitutive expression of a foreign protein (GFP). However, for *L. monocytogenes* Scott A, J0161, and G3982, it appeared that GFP-labeling negatively affected their growth and the µ_max_s of the GFP-labeled strains were significantly lower than those of the parental strains (0.29 h^−1^ vs. 0.37 h^−1^, 0.34 h^−1^ vs. 0.42 h^−1^, and 0.35 h^−1^ vs. 0.40 h^−1^, respectively). These GFP-labeled strains entered the stationary phase approximately 1 h later than their parental strains. This may indicate the trade-off between segregational stability and metabolic burden as these three strains of *L. monocytogenes* exhibited higher GFP-label stability than other strains of the same species.

In summary, *E.coli* O157:H7, *Salmonella* and *Listeria* strains can be effectively labeled with the plasmid-borne *gfp* gene which, in certain isolates, can be stable for many generations without adversely affecting growth rates. The best procedure for introducing the *gfp* gene into *E. coli* O157:H7 and *Salmonella* was the CaCl_2_ method, whereas electrotransformation was best for *Listeria* species.
